# Supplementary material for: Pyruvate Dehydrogenase Kinase 1 inhibition mediated oxidative phosphorylation enhancement in cartilage promotes osteoarthritis progression
Source: BMC Musculoskelet Disord. 2023 Jul 20;24:597. doi: 10.1186/s12891-023-06585-6 (PMC10357736; doi:10.1186/s12891-023-06585-6)
Supplement: Supplementary file 1 — Additional file 1. [file 12891_2023_6585_MOESM1_ESM.zip › BP.Gsea.1653623667859/GOBP_NEGATIVE_REGULATION_OF_RELEASE_OF_CYTOCHROME_C_FROM_MITOCHONDRIA.html]

Details for gene set GOBP\_NEGATIVE\_REGULATION\_OF\_RELEASE\_OF\_CYTOCHROME\_C\_FROM\_MITOCHONDRIA[GSEA]

|  || Dataset | 55457\_collapsed\_to\_symbols.cls.cls #OA\_versus\_normal.cls.cls #OA\_versus\_normal\_repos |
| Phenotype | cls.cls#OA\_versus\_normal\_repos |
| Upregulated in class | OA |
| GeneSet | GOBP\_NEGATIVE\_REGULATION\_OF\_RELEASE\_OF\_CYTOCHROME\_C\_FROM\_MITOCHONDRIA |
| Enrichment Score (ES) | 0.5754599 |
| Normalized Enrichment Score (NES) | 1.7942944 |
| Nominal p-value | 0.002444988 |
| FDR q-value | 0.08526671 |
| FWER p-Value | 0.986 |
Table: GSEA Results Summary

  

Fig 1: Enrichment plot: GOBP\_NEGATIVE\_REGULATION\_OF\_RELEASE\_OF\_CYTOCHROME\_C\_FROM\_MITOCHONDRIA      
 Profile of the Running ES Score & Positions of GeneSet Members on the Rank Ordered List

  

| SYMBOL | TITLE | RANK IN GENE LIST | RANK METRIC SCORE | RUNNING ES | CORE ENRICHMENT || 1 | PARL | presenilin associated rhomboid like [Source:HGNC Symbol;Acc:HGNC:18253] | 14 | 1.156 | 0.1739 | Yes |
| 2 | PSMD10 | "proteasome 26S subunit, non-ATPase 10 [Source:HGNC Symbol;Acc:HGNC:9555]" | 58 | 0.976 | 0.3183 | Yes |
| 3 | GHITM | growth hormone inducible transmembrane protein [Source:HGNC Symbol;Acc:HGNC:17281] | 213 | 0.809 | 0.4289 | Yes |
| 4 | AKT1 | AKT serine/threonine kinase 1 [Source:HGNC Symbol;Acc:HGNC:391] | 1192 | 0.478 | 0.4253 | Yes |
| 5 | PRKN | parkin RBR E3 ubiquitin protein ligase [Source:HGNC Symbol;Acc:HGNC:8607] | 1972 | 0.364 | 0.4200 | Yes |
| 6 | CLU | clusterin [Source:HGNC Symbol;Acc:HGNC:2095] | 2083 | 0.352 | 0.4647 | Yes |
| 7 | TRIAP1 | TP53 regulated inhibitor of apoptosis 1 [Source:HGNC Symbol;Acc:HGNC:26937] | 2163 | 0.342 | 0.5104 | Yes |
| 8 | IGF1 | insulin like growth factor 1 [Source:HGNC Symbol;Acc:HGNC:5464] | 2286 | 0.328 | 0.5506 | Yes |
| 9 | HGF | hepatocyte growth factor [Source:HGNC Symbol;Acc:HGNC:4893] | 2547 | 0.297 | 0.5755 | Yes |
| 10 | BCL2L2 | BCL2 like 2 [Source:HGNC Symbol;Acc:HGNC:995] | 5643 | 0.020 | 0.3383 | No |
| 11 | BCL2L1 | BCL2 like 1 [Source:HGNC Symbol;Acc:HGNC:992] | 6698 | -0.055 | 0.2649 | No |
| 12 | AVP | arginine vasopressin [Source:HGNC Symbol;Acc:HGNC:894] | 7201 | -0.096 | 0.2404 | No |
| 13 | ARRB2 | arrestin beta 2 [Source:HGNC Symbol;Acc:HGNC:712] | 7255 | -0.100 | 0.2514 | No |
| 14 | NOL3 | nucleolar protein 3 [Source:HGNC Symbol;Acc:HGNC:7869] | 7545 | -0.122 | 0.2474 | No |
| 15 | FXN | frataxin [Source:HGNC Symbol;Acc:HGNC:3951] | 8057 | -0.160 | 0.2320 | No |
| 16 | OPA1 | OPA1 mitochondrial dynamin like GTPase [Source:HGNC Symbol;Acc:HGNC:8140] | 8655 | -0.205 | 0.2166 | No |
| 17 | PPIF | peptidylprolyl isomerase F [Source:HGNC Symbol;Acc:HGNC:9259] | 9999 | -0.321 | 0.1609 | No |
| 18 | LMNA | lamin A/C [Source:HGNC Symbol;Acc:HGNC:6636] | 10946 | -0.424 | 0.1517 | No |
Table: GSEA details [plain text format]

  

Fig 2: GOBP\_NEGATIVE\_REGULATION\_OF\_RELEASE\_OF\_CYTOCHROME\_C\_FROM\_MITOCHONDRIA      
 Blue-Pink O' Gram in the Space of the Analyzed GeneSet

  

Fig 3: GOBP\_NEGATIVE\_REGULATION\_OF\_RELEASE\_OF\_CYTOCHROME\_C\_FROM\_MITOCHONDRIA: Random ES distribution      
 Gene set null distribution of ES for **GOBP\_NEGATIVE\_REGULATION\_OF\_RELEASE\_OF\_CYTOCHROME\_C\_FROM\_MITOCHONDRIA**
